# Racial and Ethnic Differences in the Association of Low-Carbohydrate Diet With Mortality in the Multi-Ethnic Study of Atherosclerosis

**DOI:** 10.1001/jamanetworkopen.2022.37552

**Published:** 2022-10-20

**Authors:** Seung-Won Oh, Alexis C. Wood, Seung-sik Hwang, Matthew Allison

**Affiliations:** 1 Department of Family Medicine, Seoul National University College of Medicine, Seoul, Republic of Korea; 2 Healthcare System Gangnam Center, Seoul National University Hospital, Seoul, Republic of Korea; 3 Department of Pediatrics, US Department of Agriculture/Agricultural Research Service Children’s Nutrition Research Center, Baylor College of Medicine, Houston, Texas; 4 Department of Public Health Sciences, Graduate School of Public Health, Seoul National University, Seoul, Republic of Korea; 5 Department of Family Medicine, University of California, San Diego

## Abstract

**Importance:**

Evidence linking low-carbohydrate diets (LCDs) to mortality is limited among multiethnic populations.

**Objective:**

To evaluate the association between LCDs and mortality by race and ethnicity.

**Design, Setting, and Participants:**

The Multi-Ethnic Study of Atherosclerosis is a large, population-based prospective cohort study of adults aged 45 to 84 years recruited from 6 US communities. A total of 6109 participants without baseline cardiovascular disease were included in this analysis. Baseline data were collected from July 2000 to August 2002, with follow-up completed by December 2017. The data were analyzed between May 2021 and April 2022.

**Exposures:**

Food frequency questionnaires were used to assess dietary intake. From these data, overall LCD scores were calculated from the percentages of energy intake from carbohydrates, fats, and proteins. Animal-based (emphasizing saturated fat and animal protein) and vegetable-based (emphasizing monounsaturated fat and vegetable protein) LCD scores were also calculated.

**Main Outcomes and Measures:**

All-cause and cause-specific mortality over a median follow-up of 15.9 years (IQR, 14.3-16.6 years).

**Results:**

Among the 6109 participants (mean [SD] age, 62.3 [10.3] years; 3190 women [52.2%]; 1623 African American [26.6%], 701 Chinese American [11.5%], 1350 Hispanic [22.1%], and 2435 non-Hispanic White [39.8%]), there were 1391 deaths. Overall, LCD scores were not associated with mortality, but when analyses were stratified by race and ethnicity, a lower risk of all-cause and non-CV mortality was observed among Hispanic participants with moderate carbohydrate intake. Specifically, the hazard ratios for total mortality for overall LCD scores were 0.58 (95% CI, 0.40-0.84) for quintile 2, 0.67 (95% CI, 0.45-0.98) for quintile 3, 0.60 (95% CI, 0.41-0.87) for quintile 4, and 0.83 (95% CI, 0.57-1.21) for quintile 5, with quintile 1 as the reference group. A similar association was observed for animal-based (but not vegetable-based) LCD scores. In contrast, no significant associations were found between LCD scores and mortality risk in the other racial and ethnic groups.

**Conclusions and Relevance:**

In this cohort study of multiethnic US adults, LCD score was not associated with mortality in the whole population. However, moderate carbohydrate intake was associated with a lower risk of mortality among Hispanic participants. These findings suggest that the association between carbohydrate intake and mortality may differ according to race and ethnicity. Dietary guidelines considering these differences may be necessary.

## Key Points

**Question:**

Is there a difference in the association between low-carbohydrate diets and mortality among different racial and ethnic groups?

**Findings:**

In this cohort study of 6109 multiethnic participants, low-carbohydrate diets were not associated with total mortality. However, moderate carbohydrate intake was associated with a lower risk of mortality than high carbohydrate intake among Hispanic participants but not among other racial and ethnic groups.

**Meaning:**

The association between carbohydrate intake and mortality may differ according to race and ethnicity, and dietary guidelines considering these differences may be necessary.

## Teaser

This cohort study uses data from the Multi-Ethnic Study of Atherosclerosis to evaluate the association of low-carbohydrate diets with mortality in different US racial and ethnic populations.

## Introduction

Low-carbohydrate diets (LCDs), which are high in protein and fat, are reported to be effective in promoting weight loss and reducing cardiometabolic risk.^1,2^ However, LCDs can lead to an increased consumption of animal protein and saturated fat, causing potentially unfavorable changes in serum lipid levels and increasing the risk of cardiovascular (CV) disease and cancer. Therefore, the long-term effects and safety of LCDs remain controversial.^3,4^

Several large cohort studies and meta-analyses have revealed a link between LCD and mortality.^5,6,7,8^ In the Nurses’ Health Study and Health Professionals Follow-up Study, animal-based LCDs were associated with higher all-cause mortality. In contrast, vegetable-based LCDs were associated with lower all-cause and CV mortality rates.^9^ Another cohort study and meta-analysis reported similar results.^10,11^ These findings suggest that the association between LCD and mortality could differ according to the source of macronutrients.

In contrast to Western studies, a Japanese study reported that LCDs were inversely associated with CV and all-cause mortality, which may be attributable to relatively low intakes of animal fat and protein in Japan.^12^ Moreover, the Prospective Urban Rural Epidemiology (PURE) study of 18 countries across 5 continents reported that high-carbohydrate intake was associated with an increased risk of all-cause and non-CV mortality.^13^ In another Japanese cohort study, both LCDs and high-carbohydrate diets were associated with higher mortality risk than moderate carbohydrate intake.^14^ Considering these conflicting results, dietary differences among racial and ethnic groups may also contribute to the association between macronutrient intake and mortality.

Hispanic populations have a lower intake of total fat and a higher intake of protein and dietary fiber than non-Hispanic Black or White populations.^15^ Indeed, diet habits are cited as one of the causes of the so-called Hispanic paradox, which combines a high prevalence of CV disease risk factors and a low mortality rate.^16^ However, no studies have confirmed the relationship between macronutrient intake and mortality in Hispanic populations or investigated the differences among diverse racial and ethnic groups. Therefore, this study aimed to assess the association between LCDs and mortality among Hispanic and non-Hispanic populations.

## Methods

### Study Population

This cohort study used data from the Multi-Ethnic Study of Atherosclerosis (MESA),^17^ a population-based study of 6814 adults aged 45 to 84 years without clinical CV disease at baseline. The cohort included 4 racial and ethnic groups from 6 communities in the US: African American (1891 [28%]), Chinese American (804 [12%]), Hispanic (1496 [22%]), and non-Hispanic White (2623 [38%]). Participants were recruited randomly or along geographic boundaries according to the field centers, ensuring a balanced distribution of age, sex, and race and ethnicity. Participants selected their race and ethnicity from the MESA screening interview questionnaire. According to MESA protocol, the surveyed racial and ethnic categories were based on the substantial proportion of previously understudied minority groups whose prevalence of risk factors and coronary heart disease risk has been shown or hypothesized to differ from the majority population. After excluding 705 individuals who provided insufficient or implausible dietary information (consumption of >6000 kcal/d or <600 kcal/d), data from 6109 participants were included in the final analysis. Ethics approval was obtained from the institutional review boards of all MESA participating centers, and written informed consent was obtained from all participants.^17^ The study followed the Strengthening the Reporting of Observational Studies in Epidemiology (STROBE) reporting guideline.^18^

### Assessment of LCD Scores

During the baseline examination period (July 2000 to August 2002), participants completed a 120-item food-frequency questionnaire (FFQ) that has been validated among African American, Hispanic, and non-Hispanic White populations and modified with additional questions to better capture food intake in the Chinese American population.^19^ Processing errors relative to the input of food frequency variables were corrected by reentering all available forms and imputing frequency and serving size values for participants whose original records no longer existed. Daily nutrient intake was calculated by multiplying the frequency and serving size for each food and beverage. Carbohydrate, fat (total, saturated, monounsaturated, and polyunsaturated), and protein (total, animal, and vegetable) intake was estimated using a dietary analysis program (Nutrition Data Systems for Research; Nutrition Coordinating Center, University of Minnesota).

The computation of the LCD scores has been previously described in detail.^20^ Briefly, we divided the participants into 11 strata, with fat, protein, and carbohydrate intake expressed as a percentage of total energy intake. For fat and protein intake, participants in the highest stratum received 10 points; those in the second-highest stratum received 9 points, and so on down to participants in the lowest stratum, who received 0 points. The lowest stratum received 10 points for carbohydrates, and the highest received 0 points. Three LCD scores were obtained. The overall LCD score was calculated as the sum of the points for total carbohydrate, fat, and protein. The animal-based and vegetable-based LCD scores were calculated as the sum of the points for carbohydrate, saturated fat, and animal protein and for carbohydrate, monounsaturated fat, and vegetable protein, respectively.

### Ascertainment of Mortality

After enrollment in MESA, all participants were contacted by phone every 9 to 12 months for information about any medical event, including interim hospital admissions, outpatient CV diagnoses and procedures, and death. If the participant died, the next of kin provided information regarding the date and cause of death, and a physician adjudication committee determined whether the cause of death was CV related (ie, myocardial infarction, coronary heart disease, stroke, other atherosclerotic diseases, or other CV diseases). Underlying causes for other deaths were obtained through the state or city vital statistics departments and the National Death Index. Follow-up was calculated as the time between enrollment and death, loss to follow-up, or end of the study period. The most current data set reported cumulative clinical events and surveillance times through the end of calendar year 2017.

### Additional Measurements

Information on demographic characteristics, lifestyle risk factors, and medical conditions was obtained using interviewer-administered questionnaires at baseline. Educational levels were categorized as less than high school graduate, high school graduate, General Educational Development, and some college or more. Health insurance was divided into 2 groups (yes or no). Physical activity in metabolic equivalent tasks was estimated using a semiquantitative questionnaire. Laboratory data and anthropometric measurements were determined as previously described.^17^ Hypertension was defined as systolic blood pressure 140 mm Hg or higher, diastolic blood pressure 90 mm Hg or higher, or medication use for hypertension.^21^ Diabetes was defined as a fasting blood glucose level of 126 mg/dL or higher or the use of glucose-lowering agents.^22^

### Statistical Analysis

The data analysis for this study was performed between May 2021 and April 2022. The participants were divided into quintiles of overall, animal-based, or vegetable-based LCD scores. We used Cox proportional hazards regression to calculate adjusted hazard ratios (HRs) and 95% CIs of total and cause-specific mortality according to quintiles of each LCD score, taking the lowest category as a reference. In the first model, we adjusted for age, sex, and race and ethnicity. In the second model, we further adjusted for education; health insurance; body mass index (BMI; calculated as weight in kilograms divided by height in meters squared); waist circumference; smoking; alcohol; physical activity (moderate and vigorous); history of hypertension, diabetes, or cancer; low-density lipoprotein and high-density lipoprotein cholesterol; and total calorie intake (obtained at baseline). We tested for potential nonlinearity using the likelihood ratio test.

Subsequently, we conducted the same analyses in subgroups based on race and ethnicity. Specifically, the interaction between LCD scores and race and ethnicity on mortality risk was investigated using additive and multiplicative scales. The additive interaction between LCD scores (first vs third quintile) and race and ethnicity was assessed by calculating the relative excess risk caused by interaction (RERI)^23,24^: (HR_11_ − HR_10_ − HR_01_) + 1, where HR_11_ was the HR for both risk factors present, HR_10_ was the HR for Hispanic participants, and HR_01_ was the HR for the first quintile of the LCD score. The RERI was considered significant when the 95% CI did not contain 0. The multiplicative interaction between LCD scores and race and ethnicity on mortality was also investigated using the log-likelihood ratio test. To visually analyze the association between LCD scores and mortality in Hispanic and non-Hispanic (ie, African American, Chinese American, and non-Hispanic White) populations, we conducted a restricted cubic spline with 4 knots placed at the 5th, 35th, 65th, and 95th percentiles of the LCD score. The reference value of the LCD score for estimating HRs and 95% CIs in the restricted cubic spline curve was 17.

We conducted sensitivity analyses to test the robustness of the results. First, we excluded the participants who died during the first year of follow-up. Second, we excluded the participants with a history of cancer. We conducted all statistical analyses using Stata, version 17.0 software (StataCorp LLC). We considered a 2-tailed *P* < .05 to be significant.

## Results

### Participant Characteristics

A total of 6109 US adults (mean [SD] age, 62.3 [10.3] years; 3190 women [52.2%] and 2919 men [47.8%]; 1623 African American [26.6%]; 701 Chinese American [11.5%]; 1350 Hispanic [22.1%]; and 2435 non-Hispanic White [39.8%]) were included in the analysis. Participants with a higher overall LCD score were more likely to be younger and be smokers and to have a higher BMI and history of diabetes. The participants with a higher overall LCD score were less likely to be Hispanic (191 [18.3%]) (Table 1). This pattern was similar in both the non-Hispanic and Hispanic groups (eTable 1 in the Supplement). Hispanic compared with non-Hispanic participants were more likely to be younger (mean [SD] age, 61.4 [10.4] vs 62.5 [10.2] years) and have a higher BMI (mean [SD], 29.4 [5.0] vs 28.0 [5.5]), blood glucose level (mean [SD], 103.9 [39.7] vs 95.3 [26.8] mg/dL [to convert to mmol/L, multiply by 0.0555]), and low-density lipoprotein cholesterol level (mean [SD], 119.2 [31.8] vs 116.4 [30.7] mg/dL [to convert to mmol/L, multiply by 0.0259]) (eTable 2 in the Supplement). Hispanic participants also had higher total energy and fiber intakes and percentages of energy intake from carbohydrates and vegetable protein (eTables 2 and 3 in the Supplement). The mean (SD) percentages of energy from carbohydrates in the lowest and highest quintiles of the LCD score were 65.5% (5.0%) and 44.9% (5.5%), respectively, in the Hispanic group and 64.8% (5.2%) and 41.9% (5.4%), respectively, in the non-Hispanic group (eTable 1 in the Supplement). The mean (SD) carbohydrate intake for the entire cohort was 53.7% (8.8%) of total energy intake.

**Table 1.** Characteristics of the Participants According to Quintiles of the Low-Carbohydrate Diet (LCD) Scores

Characteristic	Mean (SD)				
	Q1	Q2	Q3	Q4	Q5
LCD score, median (IQR)	5 (3-7)	11 (10-12)	16 (15-17)	20 (19-21)	25 (24-27)
No. of deaths	328	299	249	285	230
Age, y^a^	64.2 (10.4)	62.9 (10.2)	62.3 (10.3)	61.2 (10.3)	60.4 (9.7)
Sex, No. (%)					
Female	702 (54.2)	648 (50.2)	592 (51.8)	704 (52.8)	544 (52.0)
Male	594 (45.8)	644 (49.8)	550 (48.2)	629 (47.2)	502 (48.0)
Race and ethnicity, No. (%)					
African American	408 (31.5)	291 (22.5)	253 (22.2)	365 (27.4)	306 (29.3)
Chinese American	157 (12.1)	169 (13.1)	132 (11.6)	141 (10.6)	102 (9.8)
Hispanic^a^	327 (25.2)	327 (25.3)	245 (21.5)	260 (19.5)	191 (18.3)
Non-Hispanic White	404 (31.2)	505 (39.1)	512 (44.8)	567 (42.5)	447 (42.7)
BMI^a^	27.6 (5.1)	27.8 (5.2)	28.2 (5.3)	28.7 (5.4)	29.3 (5.9)
WC, cm^a^	96.2 (13.4)	96.9 (13.7)	98.3 (14.2)	99.3 (14.4)	100.4 (15.6)
Current smoker, No. (%)^a^	108 (8.3)	142 (11.0)	132 (11.6)	213 (16.0)	177 (17.0)
Current drinker, No. (%)^a^	647 (50.1)	704 (55.0)	679 (59.9)	781 (58.8)	624 (60.1)
MVPA, MET-min/wk	5876.9 (6131.4)	5791.6 (5829.0)	5728.2 (5592.6)	5744.7 (6173.1)	5559.4 (5736.9)
Hypertension, No. (%)^a^	640 (49.4)	579 (44.8)	489 (42.8)	573 (43.0)	449 (42.9)
Diabetes, No. (%)^a^	110 (8.5)	132 (10.3)	123 (10.8)	179 (13.5)	210 (20.1)
Lipid-lowering medication^a^	256 (19.8)	217 (16.8)	167 (14.7)	190 (14.3)	164 (15.7)
Blood pressure, mm Hg					
Systolic	128.8 (22.4)	126.5 (20.2)	126.4 (21.4)	125.8 (21.5)	125.0 (20.9)
Diastolic	72.3 (10.4)	71.9 (10.0)	71.9 (9.9)	71.8 (10.5)	71.6 (10.4)
Blood glucose, mg/dL^a^	94.1 (23.1)	96.0 (29.2)	96.2 (27.9)	97.6 (30.9)	103.2 (39.3)
Lipid profile, mg/dL					
TG	129.6 (78.3)	134.3 (96.4)	132.3 (87.4)	131.1 (87.1)	130.7 (85.9)
HDL-C	51.2 (14.9)	50.8 (15.0)	50.5 (13.8)	51.3 (15.5)	50.9 (15.2)
LDL-C	116.2 (30.4)	115.8 (30.5)	118.5 (31.1)	118.1 (30.9)	116.3 (32.0)
Macronutrient intake					
Total calories^a^	1504.9 (695.5)	1631.6 (726.5)	1711.2 (790.6)	1805.6 (842.9)	1791.6 (819.5)
Protein, g^a^	49.3 (23.7)	60.1 (25.7)	66.9 (30.7)	72.8 (33.1)	82.7 (37.8)
Carbohydrates, g^a^	243.3 (112.4)	231.7 (100.9)	226.3 (104.3)	218.9 (101.5)	191.7 (92.5)
Fat, g^a^	39.5 (20.5)	50.7 (26.1)	59.2 (30.0)	69.8 (36.1)	76.4 (36.5)
Cholesterol, mg^a^	152.0 (104.4)	207.9 (119.5)	247.3 (139.2)	304.0 (168.0)	387.4 (225.0)
Fiber, g^a^	20.9 (10.6)	20.2 (9.4)	19.7 (9.6)	18.9 (9.6)	17.2 (9.2)
Macronutrients, % of energy					
Protein^a^	13.2 (2.1)	15.0 (2.7)	15.9 (2.9)	16.5 (2.8)	18.7 (2.7)
Carbohydrates^a^	65.0 (5.1)	57.2 (4.8)	53.1 (3.5)	48.8 (3.9)	42.6 (5.5)
Fat^a^	23.4 (4.2)	27.5 (4.5)	30.8 (4.2)	34.4 (4.8)	38.3 (4.8)
Animal protein^a^	6.9 (2.1)	9.0 (2.5)	10.2 (2.7)	11.0 (2.6)	13.7 (2.9)
Vegetable protein^a^	6.2 (1.6)	5.9 (1.7)	5.6 (1.5)	5.3 (1.5)	4.9 (1.3)
Saturated fatty acids^a^	7.3 (2.0)	8.9 (2.2)	10.1 (2.3)	11.5 (2.5)	13.2 (3.0)
Monounsaturated fatty acids^a^	9.0 (1.9)	10.6 (1.9)	11.9 (1.9)	13.3 (2.1)	14.9 (2.1)
Polyunsaturated fatty acids^a^	5.0 (1.4)	5.6 (1.6)	6.1 (1.6)	6.6 (1.9)	6.8 (1.6)
Trans-fatty acids^a^	0.7 (0.3)	0.8 (0.3)	0.8 (0.3)	0.9 (0.3)	1.0 (0.3)

Abbreviations: BMI, body mass index (calculated as weight in kilograms divided by height in meters squared); HDL-C, high-density lipoprotein cholesterol; LDL-C, low-density lipoprotein cholesterol; MET, metabolic equivalent task; MVPA, moderate and vigorous physical activity; Q, quintile; TG, triglyceride; WC, waist circumference.

SI conversions: To convert blood glucose to mmol/L, multiply by 0.0555; to convert HDL-C and LDL-C to mmol/L, multiply by 0.0259; to convert TG to mmol/L, multiply by 0.0113.

^a^ Significant at *P* < .05.

### LCD Scores and Mortality

During a median follow-up of 15.9 years (IQR, 14.3-16.6 years), 1391 deaths occurred, including 328 CV deaths and 1002 non-CV deaths. After adjusting for age, sex, and race and ethnicity, the HR of all-cause mortality, comparing the highest and lowest quintiles of the overall LCD score, was 1.26 (95% CI, 1.06-1.50). For the animal-based LCD score, the HRs of all-cause and non-CV mortality of the highest quintile group were 1.30 (95% CI, 1.11-1.53) and 1.29 (95% CI, 1.07-1.56), respectively. This association became statistically nonsignificant with further adjustment (Table 2).

**Table 2.** Association of Mortality With Low-Carbohydrate Diet (LCD) Scores in the Whole Cohort^a^

Model	Adjusted HR (95% CI)				
	Q1	Q2	Q3	Q4	Q5
**Overall LCD scores**					
All-cause mortality					
No. of deaths	328	299	249	285	230
Model 1	1 [Reference]	1.08 (0.92-1.27)	1.02 (0.86-1.20)	1.10 (0.94-1.30)	1.26 (1.06-1.50)^b^
Model 2	1 [Reference]	1.00 (0.85-1.17)	0.96 (0.81-1.13)	0.96 (0.81-1.13)	1.08 (0.91-1.30)
CV mortality					
No. of deaths	79	74	55	61	59
Model 1	1 [Reference]	1.14 (0.83-1.57)	0.97 (0.69-1.38)	1.04 (0.74-1.46)	1.44 (1.02-2.03)^b^
Model 2	1 [Reference]	1.06 (0.77-1.47)	0.92 (0.65-1.31)	0.86 (0.60-1.22)	1.15 (0.80-1.65)
Non-CV mortality					
No. of deaths	234	208	183	215	162
Model 1	1 [Reference]	1.04 (0.86-1.26)	1.03 (0.85-1.26)	1.14 (0.95-1.38)	1.21 (0.99-1.49)
Model 2	1 [Reference]	0.97 (0.80-1.17)	0.97 (0.80-1.18)	1.00 (0.83-1.22)	1.06 (0.86-1.31)
**Animal-based LCD scores**					
All-cause mortality					
No. of deaths	346	282	247	240	276
Model 1	1 [Reference]	1.08 (0.92-1.26)	1.06 (0.90-1.25)	1.00 (0.85-1.18)	1.30 (1.11-1.53)^b^
Model 2	1 [Reference]	1.00 (0.85-1.17)	0.98 (0.83-1.16)	0.87 (0.73-1.03)	1.09 (0.92-1.29)
CV mortality					
No. of deaths	81	67	63	55	62
Model 1	1 [Reference]	1.12 (0.81-1.55)	1.20 (0.86-1.67)	1.05 (0.74-1.49)	1.35 (0.96-1.89)
Model 2	1 [Reference]	1.03 (0.74-1.44)	1.11 (0.79-1.55)	0.90 (0.63-1.28)	1.07 (0.75-1.52)
Non-CV mortality					
No. of deaths	247	201	173	180	201
Model 1	1 [Reference]	1.07 (0.89-1.29)	1.03 (0.85-1.25)	1.03 (0.85-1.25)	1.29 (1.07-1.56)^b^
Model 2	1	1.00 (0.82-1.20)	0.94 (0.77-1.15)	0.89 (0.73-1.09)	1.10 (0.90-1.34)
**Vegetable-based LCD scores**					
All-cause mortality					
No. of deaths	339	329	264	234	225
Model 1	1 [Reference]	0.98 (0.84-1.14)	1.10 (0.94-1.30)	1.03 (0.87-1.22)	1.06 (0.90-1.26)
Model 2	1 [Reference]	0.89 (0.77-1.04)	1.02 (0.87-1.20)	0.93 (0.79-1.11)	0.93 (0.78-1.11)
CV mortality					
No. of deaths	85	70	61	57	55
Model 1	1 [Reference]	0.84 (0.61-1.16)	1.04 (0.75-1.45)	1.05 (0.75-1.47)	1.07 (0.76-1.51)
Model 2	1 [Reference]	0.74 (0.53-1.02)	0.92 (0.66-1.28)	0.92 (0.65-1.30)	0.86 (0.60-1.22)
Non-CV mortality					
No. of deaths	238	243	194	167	160
Model 1	1 [Reference]	1.03 (0.86-1.23)	1.14 (0.95-1.38)	1.03 (0.84-1.25)	1.06 (0.87-1.30)
Model 2	1 [Reference]	0.95 (0.79-1.13)	1.07 (0.88-1.30)	0.94 (0.77-1.16)	0.95 (0.77-1.17)

Abbreviations: CV, cardiovascular; HR, hazard ratio; Q, quintile.

^a^ Model 1 was adjusted for age, sex, and race and ethnicity. Model 2 was adjusted for age, sex, race and ethnicity, education, health insurance, body mass index, waist circumference, smoking, alcohol, physical activity, history of cancer, hypertension, diabetes, low- and high-density lipoprotein cholesterol, and total calorie intake.

^b^ Significant at *P* < .05.

### Subgroup and Sensitivity Analyses

We found a significant interaction on the additive scale among ethnicity (non-Hispanic vs Hispanic, RERI, 0.37; 95% CI, 0.02-0.72), race (African American vs Hispanic, RERI, 0.42; 95% CI, 0.07-0.76), and LCD score on mortality (eTable 4 in the Supplement). Given these findings, we performed analyses by racial and ethnic group. In the Hispanic group, after full adjustment, the HRs of all-cause mortality, compared with the lowest quintile, for the second lowest through the highest quintiles of the score were 0.58 (95% CI, 0.40-0.84), 0.67 (95% CI, 0.45-0.98), 0.60 (95% CI, 0.41-0.87), and 0.83 (95% CI, 0.57-1.21). There was no significant association between overall LCD score and mortality in the non-Hispanic group (Table 3). This difference between groups was also observed in the restricted cubic spline model. A nonlinear U-shaped association was found in the Hispanic group only (Figure). This U-shaped association was observed for non-CV mortality but not for CV mortality risk. The animal-based LCD score was also nonlinearly associated with all-cause and non-CV mortality risk in the Hispanic group only (Table 3). However, no clear association was found between vegetable-based LCD scores and CV and non-CV mortality in all groups. Further subgroup analysis by race among non-Hispanic participants revealed no significant associations between LCD score and mortality risk in the African American, Chinese American, and non-Hispanic White populations (eTable 5 in the Supplement). In sensitivity analyses, the association in the Hispanic group remained similar when we excluded deaths during the first-year follow-up or participants with cancer at baseline (eTable 6 in the Supplement).

**Table 3.** Association of Mortality With Low-Carbohydrate Diet (LCD) Scores by Ethnicity^a^

Ethnicity	Adjusted HR (95% CI)				
	Q1	Q2	Q3	Q4	Q5
**Overall LCD scores**					
All-cause mortality					
Hispanic	1 [Reference]	0.58 (0.40-0.84)^b^	0.67 (0.45-0.98)^b^	0.60 (0.41-0.87)^b^	0.83 (0.57-1.21)
Non-Hispanic	1 [Reference]	1.14 (0.96-1.36)	1.02 (0.84-1.23)	0.99 (0.82-1.20)	1.12 (0.91-1.37)
CV mortality					
Hispanic	1 [Reference]	0.87 (0.42-1.77)	0.63 (0.27-1.44)	0.62 (0.28-1.38)	1.14 (0.54-2.42)
Non-Hispanic	1 [Reference]	1.16 (0.80-1.66)	0.97 (0.65-1.44)	0.88 (0.59-1.32)	1.17 (0.77-1.77)
Non-CV mortality					
Hispanic	1 [Reference]	0.50 (0.32-0.79)^b^	0.67 (0.43-1.05)	0.56 (0.36-0.88)^b^	0.72 (0.46-1.13)
Non-Hispanic	1 [Reference]	1.15 (0.93-1.42)	1.05 (0.84-1.32)	1.05 (0.85-1.31)	1.13 (0.89-1.44)
**Animal-based LCD scores**					
All-cause mortality					
Hispanic	1 [Reference]	0.72 (0.50-1.02)	0.80 (0.54-1.17)	0.50 (0.33-0.75)^b^	0.95 (0.64-1.40)
Non-Hispanic	1 [Reference]	1.05 (0.88-1.26)	1.06 (0.88-1.28)	0.92 (0.76-1.12)	1.15 (0.96-1.39)
CV mortality					
Hispanic	1 [Reference]	0.64 (0.31-1.35)	0.99 (0.48-2.05)	0.68 (0.31-1.51)	0.88 (0.39-1.98)
Non-Hispanic	1 [Reference]	1.20 (0.83-1.73)	0.98 (0.66-1.46)	0.89 (0.59-1.34)	1.14 (0.77-1.70)
Non-CV mortality					
Hispanic	1 [Reference]	0.70 (0.46-1.06)	0.70 (0.44-1.11)	0.39 (0.24-0.65)^b^	0.92 (0.59-1.45)
Non-Hispanic	1 [Reference]	1.05 (0.85-1.30)	1.12 (0.90-1.39)	1.00 (0.80-1.25)	1.18 (0.95-1.48)
**Vegetable-based LCD scores**					
Total mortality					
Hispanic	1 [Reference]	0.81 (0.55-1.20)	0.74 (0.51-1.06)	0.85 (0.59-1.22)	0.95 (0.63-1.42)
Non-Hispanic	1 [Reference]	0.98 (0.83-1.17)	1.03 (0.86-1.24)	1.02 (0.85-1.23)	0.94 (0.77-1.15)
CV mortality					
Hispanic	1 [Reference]	0.46 (0.19-1.14)	0.62 (0.29-1.29)	0.98 (0.50-1.95)	1.00 (0.45-2.21)
Non-Hispanic	1 [Reference]	0.78 (0.55-1.12)	0.97 (0.67-1.41)	0.84 (0.56-1.25)	0.88 (0.59-1.33)
Non-CV mortality					
Hispanic	1 [Reference]	0.94 (0.60-1.48)	0.78 (0.51-1.20)	0.77 (0.49-1.20)	0.93 (0.57-1.52)
Non-Hispanic	1 [Reference]	1.07 (0.88-1.31)	1.08 (0.87-1.35)	1.10 (0.88-1.37)	0.96 (0.76-1.23)

Abbreviations: CV, cardiovascular; HR, hazard ratio; Q, quintile.

^a^ Adjusted for age, sex, education, health insurance, body mass index, waist circumference, smoking, alcohol, physical activity, history of cancer, hypertension, diabetes, low- and high-density lipoprotein cholesterol, and total calorie intake.

^b^ Significant at *P* < .05.

**Figure.**
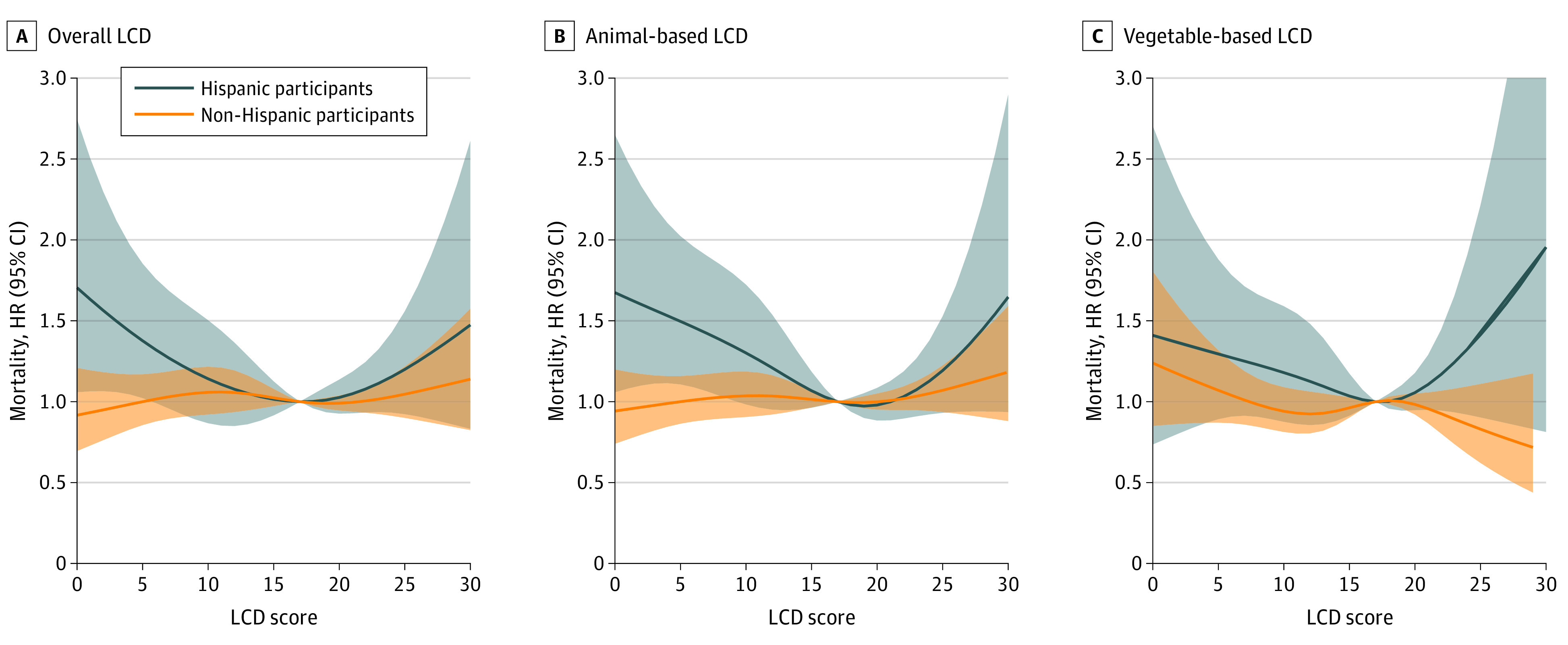
Association of Low-Carbohydrate Diet (LCD) Scores and All-Cause Mortality in Hispanic and Non-Hispanic Participants by Restricted Cubic Splines The reference value of the LCD score for estimating hazard ratios (HRs) and 95% CIs in the restricted cubic spline curve was 17. The models were adjusted for age, sex, education, health insurance, body mass index, waist circumference, smoking, alcohol, physical activity, history of cancer, hypertension, diabetes, and low- and high-density lipoprotein cholesterol. Shaded areas represent 95% CIs.

## Discussion

We observed no evidence of an independent association between LCD scores and mortality in this multiethnic, community-based population. However, the association between LCDs and mortality differed according to race and ethnicity. We observed a lower risk of all-cause and non-CV mortality in the moderate-carbohydrate-intake group among the Hispanic participants. A similar association was found in this population between animal-based LCD scores and all-cause and non-CV mortality risk.

Previous European and US cohort studies and meta-analyses found a positive association between LCD scores and all-cause mortality.^5,6,7,8^ Furthermore, LCDs based on animal sources have been associated with higher all-cause mortality, whereas vegetable-based LCDs have been associated with lower all-cause and CV mortality rates.^9,10^ However, LCD scores, based on different food sources, were not associated with total and cause-specific mortality in our study. This difference in findings could be related to the differences in participant characteristics and race and ethnicity.^24^ The MESA was based on a community-based cohort without CV disease at baseline and comprised generally older participants than cohorts examined in previous studies.^5,6,8,25^ These other studies included mainly White populations, whereas the proportion of White participants in the MESA population was less than 40%. In the National Health and Nutrition Examination Survey cohort study,^10^ subgroup analysis also showed differences according to sex and race and ethnicity, and unhealthy LCD scores were associated with high mortality, mainly in men and non-Hispanic White participants.

Studies conducted outside Europe and the US support the hypothesis of differences in the association between macronutrient intake and mortality by race and ethnicity. The NIPPON DATA80 (National Integrated Project for Prospective Observation of Non-communicable Disease and Its Trends in the Aged 1980) study^12^ reported that overall LCD scores were negatively associated with all-cause and CV mortality risk among Japanese women. The PURE study reported that high carbohydrate intake was associated with increased mortality risk and that a higher intake of total fat and various types of fat was associated with lower all-cause mortality risk.^13^ The discrepancy between studies of different racial and ethnic groups could be related to the differences in the amount and source of macronutrients. The baseline carbohydrate intake in the NIPPON DATA80 and PURE studies was approximately 60% of total energy intake, substantially higher than the 40% in Western studies.^12,13^ In our study, the mean carbohydrate intake was 53.7% of total energy intake, which was also higher than previous US studies.

Our subgroup analysis suggests that both LCDs with high animal protein and fat intake and high-carbohydrate diets with low animal protein and fat intake are associated with an increased mortality risk in the Hispanic population. Low-carbohydrate diets tend to result in a reduced intake of vegetables, fruits, and grains and a higher intake of animal protein and saturated fat, both of which have been linked to increased mortality.^26,27^ On the other hand, high-carbohydrate diets, especially those based on refined carbohydrates such as white rice, can increase glycemic load and the risk of dyslipidemia, leading to negative metabolic consequences.^28,29^ In our study, the mean carbohydrate intake in the Hispanic group was higher than that of the non-Hispanic group. In addition, the mean percentages of energy from carbohydrates in the lowest and highest quintiles of the LCD score were 65.5% and 44.9% in the Hispanic group and 64.8% and 41.9% in the non-Hispanic group, respectively. It has been reported that Hispanic people are more likely to have lower insulin sensitivity than non-Hispanic Black or White people.^30,31^ Thus, the negative effects of high carbohydrate intake on mortality may have been more evident in the Hispanic group.

A U-shaped association between the percentage of carbohydrate intake and mortality was reported in the Atherosclerosis Risk in Communities cohort.^11^ This finding was corroborated by a pooled analysis with 7 other cohort studies. In a meta-analysis, both low (<40%) and high (>70%) carbohydrate consumption conferred a greater mortality risk than a moderate carbohydrate intake.^11^ The Japan Public Health Center–based prospective study of 93 654 Japanese adults also reported a U-shaped association between overall and animal-based LCD scores and all-cause mortality.^14^

The source of carbohydrates may also contribute to racial and ethnic differences in mortality risk.^32^ A major source of carbohydrates in the Hispanic diet is rice, which constitutes only a small percentage of carbohydrate intake in non-Hispanic Black and White populations.^33^ However, a similar association was not observed among the Chinese American participants who had a high-carbohydrate intake in our study, which may be due to this population only accounting for 11% of participants. Differences in nutrient intake other than carbohydrates may also affect the outcomes. According to National Health and Nutrition Examination Survey data, the Hispanic population consumes more protein and fiber than non-Hispanic Black and White populations,^34,35^ as well as more beans and legumes than any other racial and ethnic group in the US.^36^ In our study, protein and fiber intake was also higher in the Hispanic group than in the non-Hispanic group. However, the fiber intake of the highest LCD score quintile was lower than the other 4 quintiles in the Hispanic population.

### Strengths and Limitations

To our knowledge, there have been no other studies on the association between carbohydrate intake and mortality risk in the Hispanic population. Furthermore, the use of a prospective design and data from a large, multiethnic, and community-based population increases the generalizability of our findings. The MESA had rigorous and standardized data collection procedures and extensive quality control, and the validity of the FFQ has been confirmed through various studies.^17,37^

Our study also has several limitations. First, we did not consider the heterogeneity of the Hispanic population. In a study of Hispanic people living in the US, there were differences in the intake of total fat, saturated fat, sodium, refined carbohydrates, and red meat depending on countries and regions of origin, such as Cuba, Dominican Republic, Mexico, Puerto Rico, and Latin America.^38^ Thus, studies that categorize Hispanic people into 1 group may miss differences in dietary intake that could explain differential health outcomes. Additional studies are needed to investigate the association between various dietary patterns and mortality according to different Hispanic backgrounds. Second, although the association between LCD and mortality was more pronounced for non-CV mortality than CV mortality in the Hispanic population, specific causes of death could not be identified. Several studies have reported differences in association based on cause of death; however, these results are inconsistent.^5,9,13,25^ Third, dietary intake was assessed at baseline only, and participants may have changed their diets during follow-up. Thus, misclassification of dietary intake is possible. Fourth, dietary intake is not perfectly measured by FFQ; however, measurement error in the assessment of dietary intake, and the resulting misclassification, should be nondifferentially distributed in the prospective study. Fifth, although we adjusted for various important confounders, unmeasured or unknown confounders could not be entirely excluded.

## Conclusions

We did not observe a significant independent association between LCD score and all-cause or cause-specific mortality in the overall MESA cohort. However, moderate carbohydrate intake was associated with a lower risk of mortality than high carbohydrate intake in the Hispanic population. Our findings suggest that the association between carbohydrate intake and mortality may differ according to race and ethnicity, and dietary guidelines considering these differences may be necessary.

## Notes

Supplement. **eTable 1.** Characteristics of the Participants According to Quintiles of the Low-Carbohydrate Diet Score Stratified by Ethnicity **eTable 2.** Characteristics of the Participants According to Ethnicity **eTable 3.** Characteristics of the Participants According to Race **eTable 4.** Interaction Between Low-Carbohydrate Diet Scores and Race and Ethnicity on the Total Mortality **eTable 5.** Adjusted Hazard Ratios (95% CIs) for Mortality Across Quintiles of Low-Carbohydrate Diet Score by Race **eTable 6.** Adjusted Hazard Ratios (95% CIs) for Mortality Across Quintiles of Low-Carbohydrate Diet Score, Excluding Participants Who Died During the First Year of Follow-up or Had a History of Cancer
