# Supplementary material for: Racial and Ethnic Differences in the Association of Low-Carbohydrate Diet With Mortality in the Multi-Ethnic Study of Atherosclerosis
Source: JAMA Netw Open. 2022 Oct 20;5(10):e2237552. doi: 10.1001/jamanetworkopen.2022.37552 (PMC9585425; doi:10.1001/jamanetworkopen.2022.37552)
Supplement: Supplement. — eTable 1. Characteristics of the Participants According to Quintiles of the Low-Carbohydrate Diet Score Stratified by Ethnicity eTable 2. Characteristics of the Participants According to Ethnicity eTable 3. Characteristics of the Participants According to Race eTable 4. Interaction Between Low-Carbohydrate Diet Scores and Race and Ethnicity on the Total Mortality eTable 5. Adjusted Hazard Ratios (95% CIs) for Mortality Across Quintiles of Low-Carbohydrate Diet Score by Race eTable 6. Adjusted Hazard Ratios (95% CIs) for Mortality Across Quintiles of Low-Carbohydrate Diet Score, Excluding Participants Who Died During the First Year of Follow-up or Had a History of Cancer [file jamanetwopen-e2237552-s001.pdf]

Supplemental Online Content

Oh SW, Wood AC, Hwang SS, Allison M. Racial and ethnic differences in the association of low-carbohydrate diet with mortality in the Multi-Ethnic Study of Atherosclerosis. *JAMA Netw Open*. 2022;5(10):e2237552. doi:10.1001/jamanetworkopen.2022.37552

eTable 1. Characteristics of the Participants According to Quintiles of the Low-Carbohydrate Diet Score Stratified by Ethnicity

eTable 2. Characteristics of the Participants According to Ethnicity

eTable 3. Characteristics of the Participants According to Race

eTable 4. Interaction Between Low-Carbohydrate Diet Scores and Race and Ethnicity on the Total Mortality

eTable 5. Adjusted Hazard Ratios (95% CIs) for Mortality Across Quintiles of Low-Carbohydrate Diet Score by Race

eTable 6. Adjusted Hazard Ratios (95% CIs) for Mortality Across Quintiles of Low-Carbohydrate Diet Score, Excluding Participants Who Died During the First Year of Follow-up or Had a History of Cancer

This supplemental material has been provided by the authors to give readers additional information about their work.

© 2022 Oh SW et al. *JAMA Network Open.*

eTable 1. Characteristics of the Participants According to Quintiles of the Low-Carbohydrate Diet Score Stratified by Ethnicity

	Hispanic (N=1,350)					Non-Hispanic (N=4,759)				
	Q1	Q2	Q3	Q4	Q5	Q1	Q2	Q3	Q4	Q5
LCD score, median (IQR)	5 (4)	11 (2)	15 (2)	20 (2)	26 (4)	5 (4)	11 (2)	16 (2)	20 (2)	25 (3)
Age, years	63.1 ± 10.6	62.6 ± 10.6	61.1 ± 10.2	60.4 ± 10.4	59.3 ± 9.8	64.4 ± 10.3	63.2 ± 10.1	62.7 ± 10.5	61.3 ± 10.1	60.6 ± 9.6
Women	156 (53.1)	145 (50.9)	113 (48.1)	133 (46.7)	138 (55.0)	542 (54.4)	509 (50.6)	482 (53.0)	580 (54.6)	392 (49.9)
BMI, kg/m^2	28.4 ± 4.3	28.8 ± 5.0	28.9 ± 4.4	30.3 ± 5.4	30.8 ± 5.4	27.3 ± 5.2	27.5 ± 5.3	28.0 ± 5.5	28.2 ± 5.3	29.0 ± 6.0
WC, cm	98.2 ± 11.7	99.1 ± 12.5	100.0 ± 12.0	102.8 ± 13.9	103.4 ± 13.8	95.4 ± 13.7	96.4 ± 14.1	97.6 ± 14.6	98.4 ± 14.4	99.8 ± 16.0
Current smoker	23 (7.8)	30 (10.5)	37 (15.7)	47 (16.5)	40 (15.9)	82 (8.2)	113 (11.3)	102 (11.3)	170 (16.0)	128 (16.4)
Current drinker	131 (44.6)	129 (45.3)	125 (53.2)	141 (49.7)	126 (50.2)	526 (53.0)	565 (56.8)	561 (62.3)	640 (60.4)	491 (63.1)
MVPA, MET-min/wk	5713.2 ± 5261.9	5840.3 ± 6028.2	5900.9 ± 6161.4	6872.5 ± 7335.7	5634.0 ± 5452.9	5918.7 ± 6315.2	5727.9 ± 5663.8	5645.7 ± 5495.9	5596.4 ± 5966.9	5440.5 ± 5729.7
Hypertension	135 (45.9)	122 (42.8)	102 (43.4)	111 (39.0)	93 (37.1)	509 (51.1)	454 (45.1)	396 (43.6)	458 (43.1)	350 (44.6)
Diabetes	28 (9.6)	40 (14.0)	40 (17.0)	57 (20.1)	69 (27.5)	81 (8.3)	90 (9.0)	80 (8.8)	124 (11.7)	144 (18.4)
Lipid lowering medication	44 (14.9)	50 (17.5)	26 (11.1)	25 (8.8)	26 (10.4)	212 (21.3)	175 (17.5)	141 (15.6)	164 (15.5)	131 (16.7)
SBP, mmHg	127.9 ± 21.8	129.3 ± 22.0	126.5 ± 21.5	126.8 ± 21.8	125.2 ± 22.4	129.2 ± 22.6	125.7 ± 19.9	126.3 ± 20.8	125.4 ± 21.5	125.1 ± 20.5
DBP, mmHg	72.3 ± 10.3	72.2 ± 9.6	71.8 ± 10.4	71.7 ± 10.0	70.5 ± 10.3	72.5 ± 10.6	71.5 ± 10.0	72.0 ± 9.9	71.9 ± 10.6	72.0 ± 10.2
Blood sugar, mg/dL	98.1 ± 30.8	100.5 ± 33.6	104.8 ± 39.7	103.7 ± 35.6	113.7 ± 55.1	92.8 ± 20.5	94.6 ± 27.5	93.7 ± 22.3	95.7 ± 27.5	100.9 ± 34.8
HDL, mg/dL	49.1 ± 14.1	46.8 ± 12.7	46.7 ± 12.1	46.9 ± 12.4	47.7 ± 13.4	52.2 ± 15.3	52.0 ± 15.2	51.5 ± 14.3	52.4 ± 15.8	51.6 ± 15.6
TG, mg/dL	153.1 ± 86.8	160.0 ± 88.1	155.7 ± 89.1	160.5 ± 118.0	156.2 ± 81.3	121.2 ± 73.5	126.7 ± 97.4	124.4 ± 85.4	124.2 ± 75.1	125.4 ± 86.9
LDL, mg/dL	120.3 ± 31.2	115.6 ± 30.0	123.1 ± 31.5	120.3 ± 35.0	117.0 ± 30.4	115.4 ± 30.0	114.9 ± 30.7	117.8 ± 30.3	117.9 ± 30.4	115.7 ± 32.2
Total calorie, cal	1562.5 ± 743.1	1635.5 ± 713.4	1835.0 ± 879.3	1876.2 ± 901.1	1837.4 ± 866.0	1489.0 ± 686.1	1612.7 ± 726.2	1683.4 ± 750.1	1793.9 ± 833.7	1781.6 ± 802.0
Protein, g	51.3 ± 25.1	60.9 ± 24.7	72.5 ± 35.2	77.5 ± 37.0	85.0 ± 41.1	48.8 ± 23.5	59.1 ± 25.8	65.5 ± 28.0	72.0 ± 32.2	82.4 ± 37.0
Carbohydrate, g	254.4 ± 118.4	237.0 ± 96.8	251.3 ± 119.8	239.6 ± 116.1	207.3 ± 102.4	240.1 ± 111.2	227.7 ± 101.4	220.3 ± 96.9	214.3 ± 98.2	187.7 ± 89.3
Fat, g	40.1 ± 21.7	49.4 ± 23.5	60.6 ± 30.6	68.4 ± 35.1	75.0 ± 35.6	39.4 ± 20.3	50.7 ± 26.7	59.1 ± 29.9	70.3 ± 36.5	76.9 ± 36.4
Cholesterol, mg	152.8 ± 107.0	212.2 ± 125.0	273.9 ± 164.5	313.4 ± 176.0	366.3 ± 221.0	150.6 ± 101.9	206.0 ± 116.7	241.7 ± 133.8	302.2 ± 167.1	394.4 ± 224.7

© 2022 Oh SW et al. *JAMA Network Open.*

Fiber, g	21.4 ± 11.3	20.9 ± 9.3	22.4 ± 12.1	21.5 ± 11.8	19.0 ± 11.3	20.8 ± 10.4	19.8 ± 9.4	18.9 ± 8.4	18.4 ± 8.8	16.7 ± 8.6
Macronutrients, % of energy										
Protein	13.1 ± 2.1	15.2 ± 2.5	15.9 ± 2.6	16.7 ± 2.6	18.6 ± 2.4	13.2 ± 2.1	15.0 ± 2.8	15.9 ± 2.9	16.4 ± 2.9	18.7 ± 2.8
Carbohydrate	65.5 ± 5.0	58.5 ± 3.9	55.0 ± 2.8	51.1 ± 3.1	44.9 ± 5.5	64.8 ± 5.2	56.8 ± 5.0	52.6 ± 3.5	48.2 ± 4.0	41.9 ± 5.4
Fat	22.8 ± 3.7	27.0 ± 3.9	29.6 ± 3.5	32.5 ± 4.2	36.9 ± 4.9	23.6 ± 4.3	27.8 ± 4.6	31.2 ± 4.4	34.8 ± 4.8	38.7 ± 4.7
Animal protein	6.96 ± 2.15	9.05 ± 2.56	10.03 ± 2.60	11.00 ± 2.55	13.46 ± 2.82	6.86 ± 2.11	8.96 ± 2.48	10.18 ± 2.72	10.99 ± 2.60	13.75 ± 2.92
Vegetable protein	6.10 ± 1.65	6.02 ± 1.60	5.80 ± 1.50	5.61 ± 1.45	5.05 ± 1.45	6.21 ± 1.59	5.87 ± 1.68	5.58 ± 1.53	5.28 ± 1.45	4.83 ± 1.28
Saturated fatty acid	7.61 ± 2.01	9.26 ± 2.16	10.25 ± 1.99	11.49 ± 2.43	13.64 ± 3.12	7.11 ± 2.00	8.73 ± 2.22	10.14 ± 2.34	11.43 ± 2.53	13.16 ± 2.82
Monounsaturated fatty acid	8.72 ± 1.68	10.33 ± 1.77	11.32 ± 1.62	12.48 ± 1.82	14.15 ± 2.11	9.06 ± 1.92	10.70 ± 1.98	12.05 ± 1.92	13.50 ± 2.07	15.13 ± 2.09
Polyunsaturated fatty acid	4.41 ± 1.07	4.95 ± 1.34	5.46 ± 1.63	5.73 ± 1.67	5.89 ± 1.63	5.19 ± 1.42	5.84 ± 1.57	6.24 ± 1.52	6.87 ± 1.86	6.98 ± 1.59
Trans fatty acid	0.59 ± 0.23	0.69 ± 0.25	0.74 ± 0.23	0.80 ± 0.23	0.88 ± 0.25	0.68 ± 0.30	0.77 ± 0.32	0.88 ± 0.32	0.95 ± 0.33	0.98 ± 0.32

Data are presented as number (percentage) of study participants and mean ± SD unless otherwise indicated. Abbreviations: IQR, interquartile range; BMI, body mass index; WC, waist circumference; MVPA, moderate & vigorous physical activity; SBP, systolic blood pressure; DBP, diastolic blood pressure; TG, triglyceride; HDL-C, high-density lipoprotein cholesterol; LDL-C, low-density lipoprotein cholesterol

© 2022 Oh SW et al. *JAMA Network Open.*

eTable 2. Characteristics of the Participants According to Ethnicity

	Total	Hispanic	Non-Hispanic
No. of population	6,109	1,350 (22.1)	4,759 (77.9)
No. of death	1,391	271	1,120
Age, years*	62.3 ± 10.3	61.4 ± 10.4	62.5 ± 10.2
Women	3,190 (52.2)	685 (50.7)	2,505 (52.6)
BMI, kg/m^{2}*	28.3 ± 5.4	29.4 ± 5.0	28.0 ± 5.5
WC, cm*	98.1 ± 14.3	100.6 ± 13.0	97.4 ± 14.6
Current smoker*	772 (12.7)	177 (13.1)	595 (12.5)
Current drinker*	3,435 (56.6)	652 (48.3)	2,783 (58.9)
MVPA, MET-min/wk	5747.9 ± 5911.6	6002.7 ± 6107.3	5675.5 ± 5853.5
Hypertension*	2,730 (44.7)	563 (41.7)	2,167 (45.5)
Diabetes*	754 (12.4)	234 (17.4)	520 (11.0)
Lipid lowering medication*	994 (16.3)	171 (12.7)	823 (17.3)
SBP, mmHg	126.6 ± 21.3	127.2 ± 21.9	126.4 ± 21.2
DBP, mmHg	71.9 ± 10.2	71.7 ± 10.1	72.0 ± 10.3
Blood sugar, mg/dL*	97.2 ± 30.3	103.9 ± 39.7	95.3 ± 26.8
TG, mg/dL*	131.6 ± 87.2	157.1 ± 93.9	124.3 ± 83.8
HDL-C, mg/dL*	51.0 ± 14.9	47.5 ± 13.0	52.0 ± 15.3
LDL-C, mg/dL*	117.0 ± 30.9	119.2 ± 31.8	116.4 ± 30.7
Total calorie, cal/day*	1685.0 ± 783.2	1742.7 ± 829.2	1668.6 ± 769.0
Protein, g*	65.7 ± 32.3	68.8 ± 35.1	64.8 ± 31.4
Carbohydrate, g*	223.5 ± 104.2	238.3 ± 112.0	219.3 ± 101.4
Fat, g	58.5 ± 32.9	58.1 ± 32.2	58.6 ± 33.1
Cholesterol, mg	255.1 ± 173.3	260.0 ± 178.2	253.7 ± 171.9
Fiber, g*	19.5 ± 9.8	21.0 ± 11.2	19.0 ± 9.3
Macronutrients, % of energy			
Protein	15.7 ± 3.2	15.8 ± 3.0	15.7 ± 3.2
Carbohydrate*	53.7 ± 8.8	55.3 ± 8.1	53.3 ± 8.9
Fat*	30.6 ± 6.8	29.6 ± 6.3	30.9 ± 6.9
Animal protein	10.00 ± 3.36	10.00 ± 3.32	10.00 ± 3.37
Vegetable protein*	5.62 ± 1.59	5.73 ± 1.58	5.58 ± 1.59
Saturated fatty acid*	10.07 ± 3.14	10.36 ± 3.13	9.99 ± 3.14
Monounsaturated fatty acid*	11.83 ± 2.83	11.31 ± 2.59	11.97 ± 2.87
Polyunsaturated fatty acid*	5.99 ± 1.75	5.26 ± 1.57	6.20 ± 1.74
Trans fatty acid*	0.85 ± 0.34	0.82 ± 0.33	0.74 ± 0.26

Data are presented as number (percentage) of study participants and mean ± SD unless otherwise indicated. * P-value is less than .05 between Non-hispanics and Hispanics. Abbreviations: BMI, body mass index; WC, waist circumference; MVPA, moderate & vigorous physical activity; SBP, systolic blood pressure; DBP, diastolic blood pressure; TG, triglyceride; HDL-C, high-density lipoprotein cholesterol; LDL-C, low-density lipoprotein cholesterol.

© 2022 Oh SW et al. *JAMA Network Open.*

eTable 3. Characteristics of the Participants According to Race

	African	Chinese	Hispanic	Non-Hispanic White
No. of population	1,623 (26.6)	701 (11.5)	1,350 (22.1)	2,435 (39.8)
No. of death	425	127	271	568
Age, years	62.3 ± 10.0	62.3 ± 10.4	61.4 ± 10.4	62.6 ± 10.3
Women	900 (55.5)	351 (50.1)	685 (50.7)	1,254 (51.5)
BMI, kg/m^2	30.1 ± 5.8	24.0 ± 3.2	29.4 ± 5.0	27.7 ± 5.1
WC, cm	101.0 ± 14.5	87.1 ± 9.8	100.6 ± 13.0	98.0 ± 14.4
Current smoker	287 (17.8)	40 (5.7)	177 (13.1)	268 (11.0)
Current drinker	813 (50.5)	221 (31.7)	652 (48.3)	1,749 (72.4)
MVPA, MET-min/wk	6413.7 ± 6929.8	3781.4 ± 3931.1	6002.7 ± 6107.3	5730.0 ± 5399.4
Hypertension	962 (59.3)	260 (37.1)	563 (41.7)	945 (38.8)
Diabetes	278 (17.2)	92 (13.2)	234 (17.4)	150 (6.2)
Lipid lowering medication	274 (17.0)	104 (14.8)	171 (12.7)	445 (18.3)
SBP, mmHg	131.7 ± 21.5	124.4 ± 21.6	127.2 ± 21.9	123.4 ± 20.1
DBP, mmHg	74.5 ± 10.2	72.0 ± 10.5	71.7 ± 10.1	70.2 ± 9.9
Blood sugar, mg/dL	99.5 ± 31.7	98.8 ± 27.8	103.9 ± 39.7	91.5 ± 22.0
TG, mg/dL	103.5 ± 67.3	143.1 ± 84.4	157.1 ± 93.9	132.8 ± 90.5
HDL-C, mg/dL	52.8 ± 15.4	49.3 ± 12.5	47.5 ± 13.0	52.2 ± 15.8
LDL-C, mg/dL	116.1 ± 32.3	114.8 ± 28.6	119.2 ± 31.8	117.0 ± 30.1
Total calorie, cal/day	1734.8 ± 861.5	1263.8 ± 573.0	1742.7 ± 829.2	1741.0 ± 715.3
Protein, g	65.6 ± 35.7	55.4 ± 26.3	68.8 ± 35.1	67.0 ± 29.1
Carbohydrate, g	230.6 ± 114.8	176.59 ± 80.2	238.3 ± 112.0	224.1 ± 94.1
Fat, g	62.3 ± 36.7	38.7 ± 20.9	58.1 ± 32.2	61.91 ± 31.5
Cholesterol, mg	276.5 ± 192.6	217.4 ± 143.9	260.0 ± 178.2	249.0 ± 162.2
Fiber, g	18.8 ± 9.9	16.4 ± 8.1	21.0 ± 11.2	19.9 ± 9.0
Macronutrients, % of energy				
Protein	15.1 ± 3.1	17.6 ± 3.2	15.8 ± 3.0	15.5 ± 3.1
Carbohydrate	53.9 ± 9.1	56.2 ± 7.6	55.3 ± 8.1	52.1 ± 8.8
Fat	31.7 ± 6.9	27.2 ± 5.4	29.6 ± 6.3	31.4 ± 7.0
Animal protein	9.78 ± 3.33	10.57 ± 3.59	10.00 ± 3.32	9.98 ± 3.32
Vegetable protein	5.28 ± 1.48	7.03 ± 1.55	5.73 ± 1.58	5.37 ± 1.45
Saturated fatty acid	10.11 ± 2.99	7.88 ± 2.31	10.36 ± 3.13	10.53 ± 3.20
Monounsaturated fatty acid	12.41 ± 2.94	10.50 ± 2.26	11.31 ± 2.59	12.10 ± 2.85
Polyunsaturated fatty acid	6.33 ± 1.79	6.21 ± 1.54	5.26 ± 1.57	6.11 ± 1.75
Trans fatty acid	0.95 ± 0.34	0.52 ± 0.25	0.74 ± 0.26	0.88 ± 0.30

Data are presented as number (percentage) of study participants and mean ± SD unless otherwise indicated. P-values are less than .05 in every variables. Abbreviations: BMI, body mass index; WC, waist circumference; MVPA, moderate & vigorous physical activity; SBP, systolic blood pressure; DBP, diastolic blood pressure; TG, triglyceride; HDL-C, high-density lipoprotein cholesterol; LDL-C, low-density lipoprotein cholesterol.

© 2022 Oh SW et al. *JAMA Network Open.*

eTable 4. Interaction Between Low-Carbohydrate Diet Scores and Race and Ethnicity on the Total Mortality

	Quintile 3 of LCD score		Quintile 1 of LCD score		RR (95% CI) for LCD score within strata of race/ethnicity
	Death/Total	RR (95% CI)	Death/Total	RR (95% CI)	
(A) Ethnicity					
Non-Hispanic	206/896	1.0	246/965	0.99 (0.82-1.20)<br>P=0.91	0.99 (0.82-1.20)<br>P=0.91
Hispanic	43/244	0.68 (0.48-0.97)<br>P=0.03	82/326	1.05 (0.79-1.39)<br>P=0.76	1.53 (1.05-2.24)<br>P=0.03
Interaction on additive scale: RERI (95% CI) = 0.37 (0.02-0.72), P=0.04; multiplicative scale: ratio of RRs (95% CI) = 0.44 (0.01-0.86), P=0.04					
(B) Race					
African	70/253	1.0	105/406	0.83 (0.61-1.15)<br>P=0.26	0.83 (0.61-1.15)<br>P=0.26
Chinese	22/132	0.45 (0.25-0.79)<br>P=0.005	31/156	0.51 (0.30-0.85)<br>P=0.01	1.14 (0.64-2.03)<br>P=0.66
Interaction on additive scale: RERI (95% CI) = 0.23 (-0.14-0.60), P=0.23; multiplicative scale: ratio of RRs (95% CI) = 0.31 (-0.34-0.97), P=0.35					
Hispanic	43/244	0.55 (0.36-0.84)<br>P=0.005	82/326	0.87 (0.60-1.27)<br>P=0.48	1.58 (1.08-2.32)<br>P=0.02
Interaction on additive scale: RERI (95% CI) = 0.42 (0.07-0.76), P=0.02; multiplicative scale: ratio of RRs (95% CI) = 0.56 (0.07-1.05), P=0.03					
Non- Hispanic White	114/511	0.83 (0.60-1.14)<br>P=0.25	110/403	0.79 (0.57-1.08)<br>P=0.14	0.95 (0.72-1.25)<br>P=0.70
Interaction on additive scale: RERI (95% CI) = 0.08 (-0.27-0.43), P=0.66; multiplicative scale: ratio of RRs (95% CI) = 0.08 (-0.33-0.49), P=0.71					

RRs are adjusted for age, sex, education, health insurance, BMI, WC, smoking, alcohol, physical activity, history of cancer, hypertension, diabetes, LDL-C, HDL-C, total calorie.

© 2022 Oh SW et al. *JAMA Network Open.*

eTable 5. Adjusted Hazard Ratios (95% CIs) for Mortality Across Quintiles of Low-Carbohydrate Diet Score by Race

	Q1	Q2	Q3	Q4	Q5
**Overall**					
**Total mortality**					
African	1	1.24 (0.94-1.64)	1.09 (0.80-1.49)	0.99 (0.73-1.34)	1.03 (0.74-1.42)
Chinese	1	0.82 (0.49-1.39)	0.72 (0.40-1.32)	1.39 (0.81-2.40)	1.01 (0.53-1.93)
Hispanic	1	**0.58 (0.40-0.84)**	**0.67 (0.45-0.98)**	**0.60 (0.41-0.87)**	0.83 (0.57-1.21)
Non-Hispanic White	1	1.21 (0.93-1.57)	1.11 (0.84-1.45)	0.96 (0.73-1.26)	1.25 (0.93-1.67)
**CV mortality**					
African	1	1.29 (0.76-2.20)	0.85 (0.44-1.63)	0.68 (0.36-1.31)	0.93 (0.50-1.71)
Chinese	1	0.47 (0.15-1.47)	1.17 (0.43-3.19)	1.58 (0.58-4.32)	0.94 (0.26-3.38)
Hispanic	1	0.87 (0.42-1.77)	0.63 (0.27-1.44)	0.62 (0.28-1.38)	1.14 (0.54-2.42)
Non-Hispanic White	1	1.34 (0.75-2.41)	1.14 (0.62-2.11)	0.85 (0.45-1.61)	1.51 (0.79-2.86)
**Non-CV mortality**					
African	1	1.20 (0.86-1.68)	1.19 (0.83-1.72)	1.10 (0.77-1.58)	1.05 (0.71-1.56)
Chinese	1	0.94 (0.51-1.74)	0.58 (0.27-1.26)	1.29 (0.67-2.51)	0.90 (0.40-2.02)
Hispanic	1	**0.50 (0.32-0.79)**	0.67 (0.43-1.05)	**0.56 (0.36-0.88)**	0.72 (0.46-1.13)
Non-Hispanic White	1	1.20 (0.88-1.63)	1.12 (0.82-1.53)	1.06 (0.78-1.44)	1.26 (0.90-1.77)
**Animal based**					
**Total mortality**					
African	1	1.17 (0.87-1.57)	1.13 (0.84-1.52)	0.93 (0.68-1.28)	1.04 (0.77-1.41)
Chinese	1	0.87 (0.52-1.43)	1.25 (0.73-2.14)	1.51 (0.86-2.65)	1.30 (0.65-2.62)
Hispanic	1	0.72 (0.50-1.02)	0.80 (0.54-1.17)	**0.50 (0.33-0.75)**	0.95 (0.64-1.40)
Non-Hispanic White	1	1.05 (0.80-1.36)	1.03 (0.79-1.36)	0.86 (0.65-1.13)	1.18 (0.91-1.54)
**CV mortality**					
African	1	1.07 (0.61-1.88)	0.76 (0.41-1.40)	0.54 (0.27-1.09)	0.98 (0.55-1.73)
Chinese	1	1.67 (0.69-4.09)	1.50 (0.54-4.20)	1.92 (0.66-5.63)	0.55 (0.07-4.53)
Hispanic	1	0.64 (0.31-1.35)	0.99 (0.48-2.05)	0.68 (0.31-1.51)	0.88 (0.39-1.98)
Non-Hispanic White	1	1.48 (0.82-2.68)	1.31 (0.69-2.48)	1.21 (0.65-2.25)	1.29 (0.68-2.44)
**Non-CV mortality**					
African	1	1.27 (0.89-1.82)	1.32 (0.92-1.87)	1.19 (0.82-1.71)	1.08 (0.74-1.58)

© 2022 Oh SW et al. *JAMA Network Open.*

Chinese	1	0.70 (0.38-1.32)	1.25 (0.66-2.38)	1.40 (0.70-2.80)	1.29 (0.57-2.93)
Hispanic	1	0.70 (0.46-1.06)	0.70 (0.44-1.11)	**0.39 (0.24-0.65)**	0.92 (0.59-1.45)
Non-Hispanic White	1	1.00 (0.73-1.35)	1.00 (0.73-1.37)	0.84 (0.62-1.15)	1.21 (0.90-1.64)
**Vegetable based**					
**Total mortality**					
African	1	1.10 (0.84-1.46)	0.95 (0.70-1.28)	0.94 (0.69-1.27)	0.93 (0.68-1.28)
Chinese	1	1.20 (0.69-2.09)	1.40 (0.78-2.54)	1.66 (0.92-3.03)	1.11 (0.58-2.13)
Hispanic	1	0.81 (0.55-1.20)	0.74 (0.51-1.06)	0.85 (0.59-1.22)	0.95 (0.63-1.42)
Non-Hispanic White	1	0.78 (0.61-1.00)	1.08 (0.84-1.39)	0.93 (0.71-1.22)	0.93 (0.70-1.24)
**CV mortality**					
African	1	0.93 (0.54-1.61)	0.75 (0.40-1.40)	0.74 (0.40-1.36)	0.78 (0.42-1.44)
Chinese	1	0.39 (0.12-1.33)	1.09 (0.40-3.01)	1.43 (0.51-4.01)	1.18 (0.40-3.47)
Hispanic	1	0.46 (0.19-1.14)	0.62 (0.29-1.29)	0.98 (0.50-1.95)	1.00 (0.45-2.21)
Non-Hispanic White	1	0.81 (0.47-1.41)	1.18 (0.68-2.06)	0.78 (0.41-1.48)	0.94 (0.49-1.78)
**Non-CV mortality**					
African	1	1.15 (0.83-1.61)	1.02 (0.71-1.47)	0.99 (0.69-1.43)	0.97 (0.66-1.43)
Chinese	1	1.85 (0.92-3.70)	1.75 (0.81-3.77)	1.89 (0.86-4.13)	1.08 (0.45-2.61)
Hispanic	1	0.94 (0.60-1.48)	0.78 (0.51-1.20)	0.77 (0.49-1.20)	0.93 (0.57-1.52)
Non-Hispanic White	1	0.76 (0.57-1.01)	1.07 (0.80-1.44)	0.98 (0.72-1.33)	0.97 (0.70-1.34)

Adjusted for age, sex, education, health insurance, BMI, WC, smoking, alcohol, physical activity, history of cancer, hypertension, diabetes, LDL-C, HDL-C, total calorie. Bold values represent statistically significant (P < .05).

© 2022 Oh SW et al. *JAMA Network Open.*

eTable 6. Adjusted Hazard Ratios (95% CI) for Mortality Across Quintiles of Low-Carbohydrate Diet Score, Excluding Participants Who Died During the First Year Follow-up or Had a History of Cancer

	Q1	Q2	Q3	Q4	Q5
(A) excluding deaths during the first year follow-up					
**Overall**					
Whole population	1	0.99 (0.84-1.17)	0.97 (0.82-1.14)	0.95 (0.80-1.12)	1.08 (0.90-1.29)
Hispanic	1	**0.56 (0.38-0.82)**	**0.67 (0.46-0.99)**	**0.59 (0.40-0.86)**	0.81 (0.55-1.19)
Non-Hispanic	1	1.14 (0.96-1.36)	1.02 (0.85-1.24)	0.99 (0.81-1.19)	1.12 (0.91-1.37)
**Animal based**					
Whole population	1	0.99 (0.84-1.16)	0.99 (0.83-1.17)	0.85 (0.71-1.00)	1.09 (0.92-1.30)
Hispanic	1	0.71 (0.49-1.01)	0.80 (0.54-1.18)	**0.48 (0.32-0.73)**	0.94 (0.63-1.39)
Non-Hispanic	1	1.04 (0.87-1.25)	1.07 (0.89-1.29)	0.90 (0.74-1.09)	1.16 (0.96-1.40)
**Vegetable based**					
Whole population	1	0.89 (0.76-1.04)	1.02 (0.86-1.20)	0.94 (0.79-1.12)	0.92 (0.77-1.10)
Hispanic	1	0.81 (0.55-1.21)	0.73 (0.51-1.06)	0.83 (0.57-1.19)	0.92 (0.61-1.38)
Non-Hispanic	1	0.99 (0.83-1.17)	1.04 (0.86-1.25)	1.03 (0.85-1.25)	0.93 (0.76-1.14)
(B) excluding participants with cancer at the baseline					
**Overall**					
Whole population	1	0.99 (0.83-1.17)	0.94 (0.78-1.13)	0.98 (0.82-1.18)	1.07 (0.88-1.29)
Hispanic	1	**0.55 (0.37-0.82)**	**0.65 (0.44-0.98)**	**0.59 (0.40-0.87)**	0.83 (0.56-1.24)
Non-Hispanic	1	1.13 (0.94-1.37)	0.97 (0.79-1.20)	1.03 (0.840-1.25)	1.10 (0.88-1.37)
**Animal based**					
Whole population	1	1.01 (0.85-1.20)	0.96 (0.80-1.15)	0.89 (0.74-1.07)	1.11 (0.92-1.32)
Hispanic	1	0.72 (0.50-1.05)	0.83 (0.55-1.25)	**0.52 (0.34-0.80)**	0.94 (0.62-1.42)
Non-Hispanic	1	1.08 (0.89-1.31)	1.02 (0.83-1.25)	0.97 (0.79-1.19)	1.17 (0.95-1.43)
**Vegetable based**					
Whole population	1	0.88 (0.74-1.04)	0.96 (0.80-1.15)	0.92 (0.76-1.10)	0.92 (0.67-1.11)
Hispanic	1	0.80 (0.53-1.20)	0.68 (0.46-1.00)	0.84 (0.57-1.22)	0.96 (0.64-1.46)
Non-Hispanic	1	0.94 (0.78-1.13)	0.96 (0.79-1.18)	0.97 (0.80-1.19)	0.91 (0.73-1.13)

Adjusted for age, sex, education, health insurance, BMI, WC, smoking, alcohol, physical activity, history of cancer, hypertension, diabetes, LDL-C, HDL-C, total calorie. Bold values represent statistically significant (P < .05).

© 2022 Oh SW et al. *JAMA Network Open.*
